# The clinical relevance of a polygenic risk score for type 2 diabetes mellitus in the Korean population

**DOI:** 10.1038/s41598-024-55313-0

**Published:** 2024-03-08

**Authors:** Na Yeon Kim, Haekyung Lee, Sehee Kim, Ye-Jee Kim, Hyunsuk Lee, Junhyeong Lee, Soo Heon Kwak, Seunggeun Lee

**Affiliations:** 1 Graduate School of Data Science, Seoul National University, Seoul, South Korea; 2 Division of Nephrology, Department of Internal Medicine, Soonchunhyang University Seoul Hospital, Seoul, South Korea; 3 Department of Clinical Epidemiology and Biostatistics, Asan Medical Center, Seoul, South Korea; 4 Department of Internal Medicine, Seoul National University Hospital, Seoul, South Korea; 5 Department of Translational Medicine, Seoul National University College of Medicine, Seoul, Korea; 6 Genomic Medicine Institute, Medical Research Center, Seoul National University College of Medicine, Seoul, South Korea

**Keywords:** Type 2 diabetes, Genetics

## Abstract

The clinical utility of a type 2 diabetes mellitus (T2DM) polygenic risk score (PRS) in the East Asian population remains underexplored. We aimed to examine the potential prognostic value of a T2DM PRS and assess its viability as a clinical instrument. We first established a T2DM PRS for 5490 Korean individuals using East Asian Biobank data (269,487 samples). Subsequently, we assessed the predictive capability of this T2DM PRS in a prospective longitudinal study with baseline data and data from seven additional follow-ups. Our analysis showed that the T2DM PRS could predict the transition of glucose tolerance stages from normal glucose tolerance to prediabetes and from prediabetes to T2DM. Moreover, T2DM patients in the top-decile PRS group were more likely to be treated with insulin (hazard ratio = 1.69, p value = 2.31E−02) than were those in the remaining PRS groups. T2DM PRS values were significantly high in the severe diabetes subgroup, characterized by insulin resistance and $\beta$-cell dysfunction (p value = 0.0012). The prediction models with the T2DM PRS had significantly greater Harrel’s C-indices than did corresponding models without it. By utilizing prospective longitudinal study data and extensive clinical risk factor information, our analysis provides valuable insights into the multifaceted clinical utility of the T2DM PRS.

## Introduction

Type 2 diabetes mellitus (T2DM) is a critical global health challenge. The International Diabetes Federation estimates that the prevalence of diabetes, which was 10.5% in 2021, would increase to 12.2% by 2045^1^. In Korea, the Korean National Health and Nutrition Examination Surveys^2^ showed that T2DM prevalence among adults surged from 8.9% in 2001 to 16.7% in 2020. As the estimated conversion rate from prediabetes to T2DM is up to 70%^3^, identifying individuals at high risk for prediabetes and T2DM is important because early targeted detection and intervention can prevent T2DM development and related complications, such as renal complications, heart disease, and stroke^4^.

In recent years, genome-wide association studies (GWAS) have identified a large number of genetic variants associated with the risk of T2DM^5^. By aggregating information from GWAS, a polygenic risk score (PRS) has been constructed to predict individual genetic susceptibility and is expected to enable enhanced screening and preventive therapies for T2DM and its medical complications^6^. Previous studies have shown that a PRS can identify individuals at high risk for T2DM^6–8^. However, existing PRS research has been largely limited to disease prediction using cross-sectional data. Although earlier studies have evaluated PRS using longitudinal data, only a few risk variants have been included in the calculation of PRS^9,10^. Moreover, T2DM PRS have been constructed and evaluated mostly in the European population. Therefore, this study aimed to bridge these gaps and contribute valuable insights into the prognostic capability of a T2DM PRS for the East Asian population.

Our objective was to construct and evaluate an East Asian T2DM PRS. We hypothesized that such a T2DM PRS will not only predict T2DM incidence but also glucose tolerance stage transition and T2DM severity. We first constructed an East Asian T2DM PRS using large biobank data from Korea and Japan. Second, we evaluated the performance of the T2DM PRS using prospective cohort data from the Korean Genome and Epidemiology Study (KoGES) with 16 years of follow-up. Our analysis revealed that patients in the top-decile of the T2DM PRS group had greater progression rates from nondiabetes to prediabetes and from prediabetes to T2DM. T2DM patients in the top-decile PRS group were more likely to be treated with insulin than were those in the remaining PRS groups. T2DM PRS values were significantly high in the severe diabetes subgroup. Furthermore, prediction models with the T2DM PRS had higher Harrel’s C-indices than did corresponding models without the T2DM PRS. By constructing and showing the prognostic value of the T2DM PRS, our study provides insights into its clinical utility.

## Results

### Study overview and PRS construction

**Figure 1:**
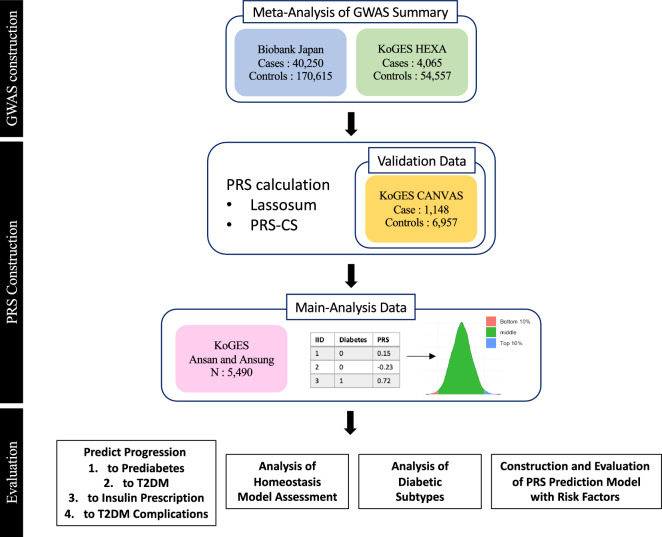
Flowchart for the PRS analysis. GWAS: genome-wide association studies, PRS: polygenic risk score; T2DM: type 2 diabetes mellitus; KoGES: Korean Genome and Epidemiology Study; HEXA: heath examinee; CANVAS: cardiovascular disease association study.

An overview of the study is provided in Fig. 1. KoGES has three cohorts: KoGES_Ansan and Ansung, KoGES_HEXA, and KoGES _CAVAS. We carried out a GWAS of T2DM using KoGES_HEXA and meta-analyzed the results with Biobank Japan T2DM GWAS results. A total of 269,487 samples (44,315 cases and 225,172 controls) were included in the meta-analysis of the East Asian T2DM GWAS summary, which was used for PRS training (Supplementary Fig. S1). KoGES_CAVAS (n = 8105) was used as validation data for hyperparameter selection. In a cross-sectional study, it is difficult to determine the predictive power of a PRS for people who have yet to develop the disease. Therefore, we used longitudinal data from KoGES Ansan and Ansung, which includes extensive follow-up for 14 years. We evaluated two genome-wide PRS construction methods, Lassosum^11^ and PRS-CS^12^.

With respect to the KoGES_Ansan and Ansung datasets, both methods performed similarly, with a slightly better area under the ROC curve (AUC) for Lassosum in the PRS-only model (Supplementary Table S1). We used the PRS constructed by Lassosum for the remainder of the study.

### Participant characteristics

A total of 5490 participants including Korean chip^13^-genotyped individuals in the KoGES_Ansan and Ansung datasets were used to evaluate the T2DM PRS. The dropout rate of the participants and the cumulative prevalence of T2DM at baseline and at each follow-up can be found in Supplementary Tables S2 and S3. At baseline, the mean age of the participants was 52 years, 47.6% were male, and 13.6% had diabetes. The participants were classified into three groups according to the PRS percentile: the top and bottom deciles and the middle (10–90%). There was no significant difference in BMI among the three PRS groups. In contrast with those in the middle and bottom decile, patients in the top-decile PRS group exhibited higher levels of low-density lipoprotein (LDL), triglycerides, fasting glucose, and HbA1c at baseline. Additionally, the top-decile PRS group had lower levels of high-density lipoprotein (HDL). T2DM prevalence in the top-decile PRS group was 8.85 times higher than that in the bottom-decile PRS group, with percentages of 32.24% and 3.64%, respectively. The top-decile PRS group also exhibited a family history of T2DM that was twice as prevalent as that of the bottom-decile PRS group. The details of the characteristics at baseline are presented in Table 1.

**Table 1:** Characteristics at the baseline of the KoGES_Ansan and Ansung dataset.

	Total (n = 5490)	PRS Bottom decile (n = 549)	Middle (10–90%) (n = 4392)	PRS Top decile (n = 549)	P-value
Basic information					
Male (%)	2614 (47.61)	267 (48.63)	2069 (47.11)	278 (50.64)	0.260
Age (year)	51.54 $\pm$ 8.50	51.63 $\pm \; 8.51$	51.50 $\pm \;8.46$	51.77 $\pm \; 8.81$	0.707
Physical measurements					
BMI (kg/m^2^)	24.63 $\pm$ 3.04	24.51 $\pm$ 3.09	24.64 $\pm \; 3.06$	24.70 $\pm \; 2.85$	0.550
SBP (mmHg)	120.87 $\pm$ 17.94	119.85 $\pm \; 17.66$	120.83 $\pm \; 18.00$	122.26 $\pm \; 17.70$	0.078
DBP (mmHg)	80.14 $\pm$ 11.22	80.01 $\pm \; 11.11$	80.10 $\pm\; 11. 23$	80.58 $\pm \;11.29$	0.620
Clinical risk factors					
HDL (mg/dL)	49.29 $\pm$ 11.52	50.01 $\pm \; 11.88$	49.32 $\pm \; 11.52$	48.39 $\pm\; 11.11$	0.063
LDL (mg/dL)	119.55 $\pm$ 33.01	117.61 $\pm \; 30.82$	119.78 $\pm \; 33.14$	119.72 $\pm \; 34.05$	0.348
TG (mg/dL)	152.90 $\pm$ 110.20	140.93 $\pm \; 96.15$	152.93 $\pm \; 111.57$	164.64 $\pm\; 111.17$	1.72E−03
Fasting glucose (mg/dL)	92.14 $\pm$ 21.14	86.58 $\pm \; 11.47$	91.65 $\pm \;19.65$	102.32 $\pm \;35.03$	< 2.00E−16
Fasting insulin ($\upmu$IU/Ml)	7.54 $\pm$ 4.48	7.23 $\pm \;3.78$	7.57 $\pm \;4.46$	7.63 $\pm \;5.21$	0.212
HbA1c (%)	5.77 $\pm$ 0.90	5.47 $\pm \; 0.48$ (36 mmol/mol)	5.75 $\pm \; 0.85$ (39 mmol/mol)	6.25 $\pm \; 1.35$ (45 mmol/mol)	< 2.00E−16
Others					
Current smoking (%)	1295 (23.59)	134 (24.42)	1025 (23.38)	136 (24.77)	0.727
Family history (%)	651 (11.86)	40 (7.29)	508 (11.57)	103 (18.76)	1.20E−08
Physical activity (%)	1981 (36.08)	190 (34.70)	1588 (36.15)	203 (36.98)	0.591
Type 2 diabetes mellitus					
Case (%)	746 (13.59)	20 (3.64)	549 (12.5)	177 (32.24)	< 2.00E−16

BMI: body mass index; WC: waist circumference; SBP: systolic blood pressure; DBP: diastolic blood pressure; HDL: high-density lipoprotein; LDL: low-density lipoprotein; TG: triglyceride. Physical Activity: people who answered that they participated in moderate to vigorous physical activity for more than 30 min daily were counted. Obtained P-value using one-way ANOVA analysis.

### Associations between the cumulative prevalence of T2DM and PRS

To investigate the relationship between the cumulative prevalence of T2DM and PRS, we conducted survival analysis with age at diagnosis as an outcome, including baseline cases. A Kaplan‒Meier plot showed that the cumulative prevalence of T2DM was significantly greater in the top PRS decile group than in the other two groups (Supplementary Fig. S2). Hazard ratios from the Cox model comparing the top and bottom-decile PRS groups with the middle PRS group were 2.29 (top vs. middle, 95% CI = 2.02–2.59, p value < 2.00E−16) and 0.45 (bottom vs. middle, 95% CI = 0.36–0.56, p value = 2.84E−13), respectively (Supplementary Table S4). In addition to the categorized PRS, we used the standardized PRS, and the hazard ratio for the latter was 1.59 (95% CI 1.52–1.67, p value < 2.00E−16).

To validate our T2DM PRS, we applied our PRS model to 1503 East Asian samples from UK Biobank (UKBB). Supplementary Fig. S3 shows that the cumulative prevalence of T2DM was significantly greater in the top-decile PRS group than in the other two groups. Hazard ratios for comparing the top-decile PRS group and bottom-decile PRS group with the middle PRS group were 2.167 (top vs. middle, 95% CI = 1.40–3.36, p value = 0.00054) and 0.36 (bottom vs. middle, 95% CI = 0.15–0.89, p value = 0.026), respectively.

### The PRS can predict incident prediabetes and T2DM

**Figure 2:**
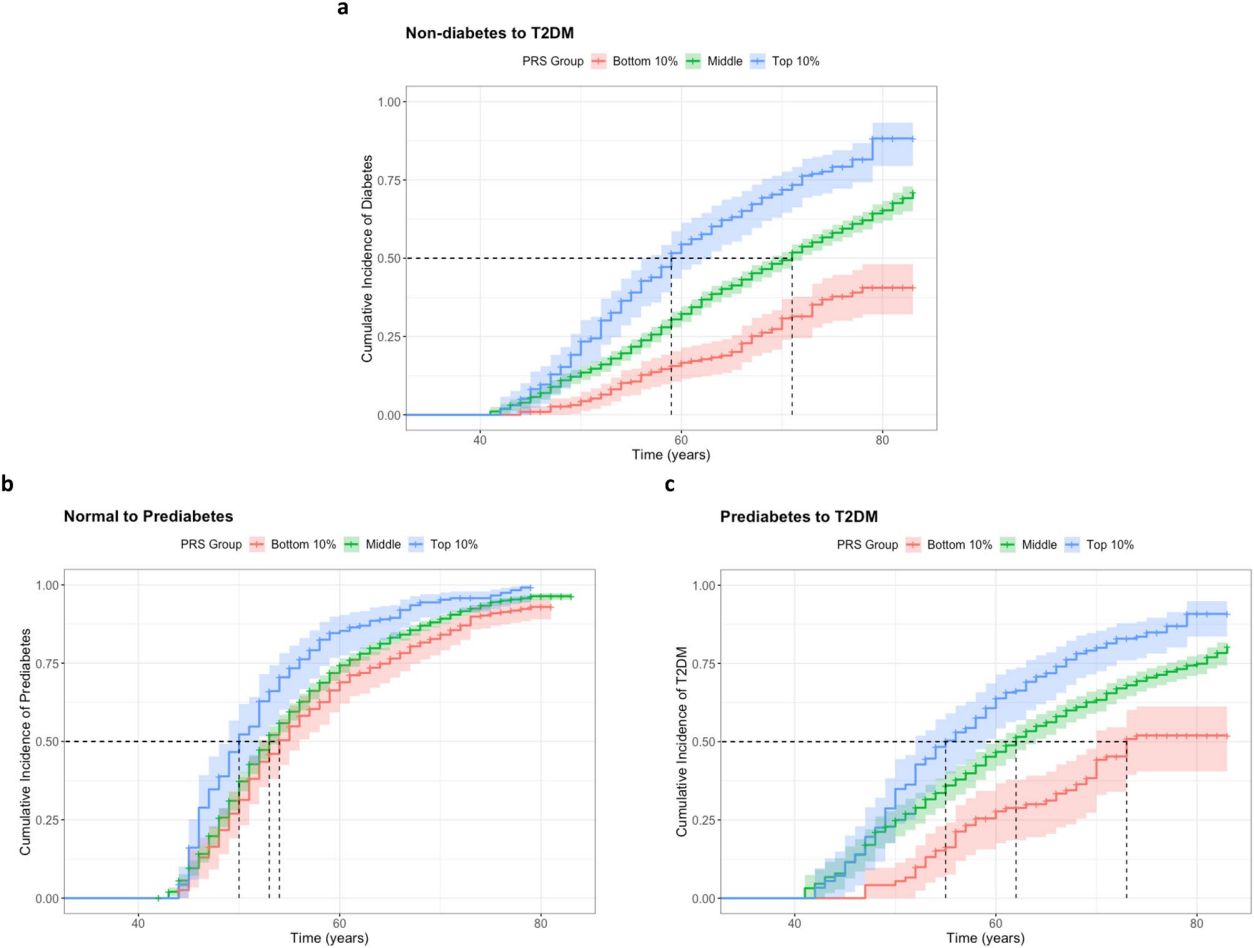
Kaplan–Meier curve for cumulative incidence of prediabetes and T2DM by PRS group. (**a**) Progression from non-diabetes to T2DM. (**b**) Progression from NGT to prediabetes. (**c**) Progression from prediabetes to T2DM. T2DM: type 2 diabetes mellitus; NGT: normal glucose tolerance; Each shaded area represents 95% confidence band for each curve. Each dash line indicates median age-at-T2DM diagnosis for each PRS group.

We hypothesized that the T2DM PRS can predict not only progression from nondiabetes to T2DM but also that from NGT to prediabetes and from prediabetes to T2DM. We included only individuals with NGT and prediabetes at baseline for the analysis. Figure 2 shows that the incidence of T2DM in both nondiabetes and prediabetes individuals was significantly greater in the top-decile PRS group than in the other two groups (p value $<$ 2.00E−16). Furthermore, the higher PRS subgroup was associated with a greater incidence of prediabetes. The hazard ratios for comparing the top-decile PRS group with the middle PRS group were 1.91 for the overall risk of T2DM in nondiabetes participants, 1.37 for progression to prediabetes from NGT, and 1.64 for progression to T2DM from prediabetes (Table 2).

**Table 2:** Results of Cox regression analysis for predicting progression from non-diabetes to T2DM, from NGT to prediabetes, and from prediabetes to T2DM.

Progression prediction	PRS	Hazard ratio	95% CI	P-value
Non-diabetes to T2DM	Categorized PRS			
	Bottom decile	0.5290	0.4161–0.6725	1.99E−07
	Middle (10–90%)	1.00 (reference)		
	Top decile	1.911	1.604–2.276	4.05E−13
	Standardized PRS	1.430	1.346–1.519	< 2.00 E−16
NGT to prediabetes	Categorized PRS			
	Bottom decile	0.8352	0.7224–0.9656	0.0150
	Middle (10–90%)	1.00 (reference)		
	Top decile	1.370	1.128–1.664	0.00151
	Standardized PRS	1.094	1.042–1.148	0.000286
Prediabetes to T2DM	Categorized PRS			
	Bottom decile	0.6181	0.4598–0.8309	0.00144
	Middle (10–90%)	1.00 (reference)		
	Top decile	1.642	1.352–2.000	6.06E−07
	Standardized PRS	1.280	1.192–1.374	1.07E−11

To evaluate the incidence cases, individuals with diabetes at the baseline were excluded. We evaluated Cox regression model with sex as a predictor. NGT: normal glucose tolerance; CI: confidence interval.

### The PRS can predict progression to insulin prescription

To assess the ability of the T2DM PRS to predict T2DM severity, we analyzed progression to insulin prescription and T2DM complications. Cox regression with PRS and sex as predictors was used to model insulin prescription. We excluded insulin-treated participants at baseline. Similarly, we used both the categorized PRS and standardized PRS. We found a significantly greater likelihood of insulin prescription among T2DM patients in the top-decile PRS group (hazard ratio = 1.69; p value = 7.61E−06). Conversely, no patient in the bottom-decile PRS group was prescribed insulin. We also fit similar models with T2DM complications as an outcome., and neither the categorized PRS nor the standardized PRS was significant in the model. The details of the results are shown in Table 3 and Supplementary Table S5.

**Table 3:** Results of Cox regression analysis for predicting insulin prescription and T2DM complications.

Prediction	PRS	Hazard ratio	95% CI	P-value
Insulin prescription	Categorized PRS			
	Remaining	1.00 (reference)		
	Top decile	1.692	1.075–2.662	0.0231
	Standardized PRS	1.663	1.331–2.077	7.61E−06
T2DM complications	Categorized PRS			
	Bottom decile	0.7841	0.4290–1.433	0.429
	Middle (10–90%)	1.00 (reference)		
	Top decile	0.8992	0.6900–1.172	0.432
	Standardized PRS	0.9878	0.8910–1.095	0.816

Cox regression model was used with sex and time from T2DM diagnosis to insulin prescription or T2DM complications in a year. For the insulin prescription prediction, participants were classified into two groups according to a percentile of PRS: top decile and remaining ($\le$ 90%). T2DM complications include myocardial infarction, coronary artery disease, congestive heart failure, cerebrovascular disease, peripheral artery disease, and kidney disease. T2DM: type 2 diabetes mellitus.

### The PRS is associated with HOMA-B

HOMA-IR and HOMA-B are biomarkers for insulin resistance and $\beta$-cell function. We investigated changes in HOMA-IR and HOMA-B in the top decile and in the remaining PRS groups during development of T2DM. For T2DM individuals, we examined the retrograde trajectories of HOMA-IR and HOMA-B by setting the diagnosis of T2DM to time zero and tracing it back every two years. For nondiabetes individuals, we examined the forward trajectories of HOMA-IR and HOMA-B from baseline. Supplementary Fig. S4 shows the changes in HOMA-IR and HOMA-B. Those who developed diabetes had an overall higher HOMA-IR and low HOMA-B than those who did not develop diabetes. As expected, we observed overall increases in HOMA-IR and decreases in HOMA-B with time closer to T2DM diagnosis. The confidence intervals of the two groups at each time point overlapped because of the small sample size. However, according to the permutation test, HOMA-B scores between the top decile and the other PRS groups were significantly different between T2DM patients (p value = 0.011) and nondiabetes patients (p value = 0.0074). We conducted the same permutation test for HOMA-IR, but there was no significant difference in HOMA-IR trajectories between the two PRS groups for diabetes (p value = 0.37) or nondiabetes (p value = 0.18).

### The PRS is associated with severe diabetic subgroup

Previous studies have suggested novel diabetic subtyping methods, which may guide prevention and treatment strategies for T2DM and its complications^14^. Therefore, we classified T2DM patients into four subgroups by data-driven k-means cluster analysis using BMI, age at diagnosis, HOMA-B, HOMA-IR, and HbA1c and observed how PRS differed within those clusters. Cluster 1 included 105 (7.94%) of the 1322 patients and was classified as a severe diabetic group with extremely high HbA1c levels, early age at diagnosis, relatively high BMI, insulin resistance (high HOMA-IR), and β-cell dysfunction (low HOMA-B). In contrast to Cluster 1, Cluster 2 (36.4%) was a mild diabetes subgroup with relatively low HbA1c and BMI and HOMA-IR and high HOMA-B. Those in Cluster 3 (30.3%), labeled mild age-related diabetes (MARD), were diagnosed with T2DM at a later age than those in other subgroups. Individuals in Cluster 4 (25.4%) had a relatively high BMI, HbA1c, and insulin resistance, and this cluster was labeled mild obesity-related diabetes (MOD). Supplementary Fig. S5 shows that even within the novel diabetic subtyping system, the PRS was significantly high in the severe diabetes subgroup (p value = 0.0012).

### The PRS can improve prospective prediction accuracy

To evaluate the improvement in prediction accuracy with the PRS, we considered a series of models with and without it. We excluded T2DM patients at baseline and used baseline measured risk factors to predict the future incidence of T2DM. The baseline model included sex and age, and we subsequently added family history, physical measurements (BMI and SBP), smoking status, and clinical risk factors (HDL, LDL, and TG). The model descriptions can be found in the Materials and Methods section. As expected, the model with a larger set of risk factors had a greater Harrel’s C-index. We also showed that models with either the standardized or categorized PRS had a significantly increased Harrel’s C-index compared with those without the PRS. For example, the Harrell’s C-index of the model with sex, age, and family history (Model 2) was 0.586, but it improved to 0.631 with the standardized PRS. We also verified that the standardized PRS was more informative for incidence prediction than was the categorized PRS by revealing a greater Harrel C-index. The details of the results are shown in Table 4 and Supplementary Table S7.

**Table 4:** Prediction performance evaluation using Harrel’s C-Index.

	Model without PRS		Model with standardized PRS		Model with categorized PRS		Model with standardized PRS vs. without PRS		Model with categorized PRS vs. without PRS	
	Harrell C	95% CI	Harrell C	95% CI	Harrell C	95% CI	C-index difference	p-value	C-index difference	p-value
Model 1	0.567	0.550–0.584	0.623	0.607–0.639	0.603	0.586–0.620	0.056	< 0.001	0.036	< 0.001
Model 2	0.586	0.569–0.603	0.631	0.615–0.647	0.613	0.596–0.630	0.045	< 0.001	0.027	< 0.001
Model 3	0.637	0.620–0.653	0.668	0.653–0.684	0.655	0.639–0.671	0.032	< 0.001	0.018	< 0.001
Model 4	0.656	0.640–0.672	0.684	0.668–0.699	0.672	0.656–0.688	0.027	< 0.001	0.016	< 0.001

Model 1: T2DM ~ sex + age; Model 2: T2DM ~ sex + age + family history; Model 3: T2DM ~ sex + age + family history + BMI + SBP + smoking status; Model 4: T2DM ~ sex + age + family history + BMI + SBP + smoking status + HDL + LDL + TG.

## Discussion

Identifying individuals at high risk for T2DM is important because early targeted detection and intervention, such as lifestyle modification or medical intervention, can delay onset or even prevent T2DM. By aggregating GWAS results, the PRS has emerged as a powerful tool for identifying individual genetic susceptibility. In addition, the PRS has the potential to be used to infer disease prognosis and subtyping^15,16^. However, current PRS research is limited primarily to disease prediction, and the clinical utility of a T2DM PRS for predicting incident T2DM has not been fully evaluated.

Our analysis demonstrated that individuals in the top-decile PRS group were more likely to experience progression to T2DM than those in the other groups. We obtained similar results when we applied more robust criteria to remove type 1 DM (T1DM) patients from the sample by excluding people aged younger than 40 years. Hazard ratios from the Cox model that compared the top and bottom deciles with the middle PRS group were 2.21 (top vs. middle, p value < 2E−16) and 0.442 (bottom vs. middle, p value = 4.11E−13), respectively.

Diagnosis of T2DM in the present study was defined by at least one criterion, as described in the Methods section. However, considering that an indicator may be temporarily high such that nondiabetes or prediabetes individuals are diagnosed with diabetes, we applied the robust criterion that more than two abnormal results obtained from the same sample constitute a diagnosis of T2DM^17,18^, and we observed similar results when we applied this robust criterion. The hazard ratio for comparison of the middle PRS group with the top-decile PRS group was 2.78 (p value < 2E−16), and that for the bottom-decile PRS group was 0.337 (p value = 2.99E−12).

Prediabetes, which is defined on the basis of glycemic parameters above the NGT but below the diabetes threshold, is a high-risk condition for diabetes with an annualized conversion rate of 5–10%^19^. Previous studies have shown that T2DM PRS is associated with prediabetes^20,21^. However, no study has shown that the T2DM PRS predicts progression from prediabetes to T2DM. Our results showed that the T2DM PRS can predict not only progression from nondiabetes to T2DM but also progression from NGT to prediabetes and from prediabetes to T2DM. By identifying high-risk individuals among the prediabetes population and providing guidelines for maintaining optimal lifestyle habits, we can reduce the progression rate from prediabetes to T2DM^22^.

A previous study showed that the T2DM PRS is a useful tool for predicting disease severity, which can be measured by escalation of treatment options and progression to T2DM complications^20^. Glucose levels of T2DM patients can be controlled by oral diabetes medication in combination with lifestyle modifications. However, some patients with a longer duration of T2DM or less well-controlled glucose levels should be treated with insulin^23^. Additionally, people with T2DM have an increased risk of developing macrovascular and microvascular complications. Our study showed that T2DM patients in the higher percentile PRS group were more likely to be prescribed insulin. However, we could not demonstrate that the T2DM PRS can predict progression to T2DM macrovascular complications or nephropathy. In previous studies, the T2DM PRS was significantly associated with an increased risk of neuropathy^20^ and cardiovascular disease^24^ but not with macrovascular complications or diabetic nephropathy. To demonstrate the association between T2DM PRS and diabetic complications, we need to further understand the biological pathway or systems that can clarify the specific cause of genetic risk and T2DM complications^25,26^.

Insulin resistance and $\beta$-cell dysfunction are used to characterize the pathophysiological mechanism of T2DM^27^, and the genetic variants linked to T2DM are associated with $\beta$-cell dysfunction^28^ and insulin secretion^29^. Previous studies have shown that *β*-cell function is impaired prior to progression from prediabetes^30^. A recent study showed that the T2DM PRS was primarily related to $\beta$-cell dysfunction in the Korean population^31^. However, the study investigated the association between the T2DM PRS and HOMA-B at baseline only. To fully understand the relationship between a PRS and HOMA-B, tracing of HOMA-B during progression to T2DM is needed. Indeed, the present study examined the trajectories of HOMA-B during development of T2DM and revealed that the HOMA-B level in the top-decile PRS group was consistently lower than that in the remaining groups, both in the group of individuals who developed diabetes and in the nondiabetes group.

Previously, diabetes was classified as type 1 or type 2 diabetes only. However, recent studies have suggested stratifying populations at risk for diabetes using clinical biomarkers to prevent progression to T2DM and even T2DM complications^14,32^. A previous study included five subgroups: severe autoimmune diabetes (SAID), severe insulin-deficient diabetes (SIDD), severe insulin-resistant diabetes (SIRD), mild obesity-related diabetes (MOD), and mild age-related diabetes (MARD)^14^. SAID was characterized by early-onset disease, relatively low BMI, high HbA1c, insulin deficiency, and glutamic acid decarboxylase antibody (GADA) presence. SIDD was similar to SAID but with GADA negative. SIDD was characterized by insulin resistance. In the present study, we classified T2DM patients into four subgroups using clustering analysis. Clusters were based on five variables that were measured at the time of diagnosis of T2DM. As the GADA test result was not provided in KoGES, we could not include GADA in the analysis. Assuming that none of the diabetes patients had autoimmune diabetes, the first cluster, named the severe diabetes subgroup, had features of a combination of SIDD and SIRD in a previous study of $\beta$-cell dysfunction and insulin resistance. However, compared to that of SIDD patients, the BMI of these patients was relatively high. The second cluster was a mild version of the first cluster. The remaining subgroups exhibited similar findings and were named the same as the MARD and MOD subgroups. We found that the PRS was also significantly high in the severe diabetes subgroup.

In our study, we found that the PRS model performed better in predicting the incidence of T2DM. The basic T2DM prediction model performed better with respect to sex, age, and PRS than without PRS. Adding family history, physical measurements, and clinical risk factors to the basic model steadily improved Harrell’s C-index. Moreover, we found evidence that use of a standardized PRS can improve prediction performance over that of the categorized PRS.

Our study has multiple strengths. First, we calculated the PRS using a recently developed method and genome-wide meta-analysis to improve the prediction accuracy. Second, by utilizing prospective longitudinal study data, we verified that the T2DM PRS is a predictor of disease risk and severity and an associated factor with the clinical biomarker HOMA-B. Moreover, we showed that the T2DM PRS is related to severe diabetes. Third, we constructed a predictive model of T2DM, including physical measurements and clinical risk factors, to increase the prediction performance. Although our analysis provides insight into the clinical utility of the T2DM PRS, there are several limitations. Our study did not include a C-peptide test or diabetes autoantibody test results, and the distinction between type 1 and type 2 diabetes among diabetes patients may be unclear. Nevertheless, we applied the robust criterion of removing T1DM patients, and the results were similar. Moreover, information on the type of oral diabetes medication or dosage of insulin prescription was not explicitly described because all questionnaires were self-reported by participants. Additionally, the participants were not asked about their history of T2DM complications but about their comprehensive history of the disease. Therefore, although we excluded participants whose incident disease was before T2DM, we cannot be sure whether those diseases were T2DM complications.

In conclusion, our analysis of prospective longitudinal study data suggests that the PRS may have clinical value. A PRS should not be considered an alternative to traditional clinical risk factors but rather a possible addition. Implementing this PRS as a clinical assessment tool can help in T2DM screening and prognosis such that complications can be prevented. Furthermore, preventive intervention and strict glycemic control may play a protective role against developing T2DM.

## Methods

KoGES is a consortium project of prospective cohort studies. The cohorts in the KoGES include the KoGES_Ansan and Ansung cohorts, the KoGES_heath examinee (HEXA) cohort, and the KoGES_cardiovascular disease association study (CANVAS), from which participants aged 40 years were recruited from the National Health Examination Registry at baseline. Due to its extensive follow-up, we used the KoGES_Ansan and Ansung studies as the main analysis data. Participants consecutively responded to the baseline and seven additional follow-up phases every two years from 2001 to 2016. Each follow-up involved administering identical questionnaires covering sociodemographic data, lifestyle, medical history, etc., physical examination (height, weight, blood pressure, etc.) and clinical investigations (blood test, urine test, etc.). The trained interviewer questioned participants’ disease history, family history of the disease, and medication prescriptions such as insulin.

### Genotyping and quality control

For our study, we utilized genotypic data that had already undergone quality control (QC) procedures by the Korea Disease Control and Prevention Agency (KDCA). The genotypes were evaluated using Korean Chip^13^. The KCDA QC protocol involved excluding samples with a low call rate (< 97%), sex discrepancies, cryptic first-degree relatives, high heterozygosity, and singletons. Genetic variants were excluded if they met the criteria of Hardy‒Weinberg equilibrium (HWE) p value (< 10E−6) or low call rate (< 95%). Genotypes were phased using Eagle v2.3 and imputed using IMPUTE4 with 1000 Genomes project phase 3 data, and the Korean reference genome was used as a reference panel. After excluding genetic variants with an imputation quality score (IQS) < 0.8 and a minor allele frequency < 1%, a total of 8,056,211 variants were used for analysis. We note that the same QC criterion was used in Nam et al.^33^, who provided a valid false positive controls.

### GWAS summary statistics construction and PRS calculation

We conducted a GWAS with 58,622 participants in the KoGES_HEXA cohort using a linear mixed model implemented in SAIGE^34^. In accordance with Nam et al.^33^, we used the age and sex of the top 10 principal components (PCs) as covariates. The top 10 PCs were used to adjust for possible population stratification. Summary statistics for Biobank Japan (BBJ) were downloaded^35^. We carried out a z-score-based meta-analysis for KoGES_HEXA with BBJ using inverse-variance weighting to obtain p values and effect sizes for risk prediction. A total of 7,057,567 variants were detected in the combined cohort.

For PRS calculation, we considered two-PRS construction methods, a penalized regression framework, Lassosum^11^, and a Bayesian regression framework, PRS-CS^12^. These two methods use GWAS summary statistics and reference panels to account for linkage disequilibrium (LD). Lassosum used an additional validation dataset for hyperparameter tuning. For the LD reference panel, we used East Asian (EAS) individuals from the 1000 Genome Project^36^. KoGES_CANVAS was used as validation data in Lassosum.

### T2DM and prediabetes

A new diagnosis of T2DM was defined by at least one of the following criteria: self-reported diagnosed diabetes, treatment with hypoglycemic medication, fasting glucose level $\ge$ 126 mg/dL, glucose level $\ge$ 200 mg/dL after the oral glucose test, or hemoglobin A1C (HbA1c) $\ge$ 6.5% (48 mmol/mol)^37^. We excluded people who were diagnosed with diabetes and aged under 30 years. According to ADA guidelines, we defined prediabetes as either a fasting glucose level of 100–125 mg/dL, a 2-h glucose level ranging from 140 mg/dL to 199 mg/dL during the 75-g oral glucose tolerance test, or an elevated HbA1c level ranging from 5.7% to 6.4% (39 to 46 mmol/mol)^23^. We defined nondiabetic individuals as individuals with normal glucose tolerance (NGT) and as individuals with prediabetes.

### T2DM complications

The participants self-reported a history of myocardial infarction, coronary artery disease, congestive heart failure, cerebrovascular disease, peripheral artery disease and kidney disease. Kidney disease was defined as a self-reported diagnosis of kidney disease or a glomerular filtration rate < 60, which was estimated with the equation suggested by Chronic Kidney Disease Epidemiology Collaboration^38^. To clarify that these diseases are T2DM complications, we excluded participants whose incident disease was ahead of T2DM in our analysis. Additionally, we used the age at diagnosis of complications as the earliest age at diagnosis.

### HOMA-IR and HOMA-B

Insulin resistance and $\beta$-cell dysfunction are important factors in understanding the pathophysiology of T2DM. HOMA-IR is a method used to measure insulin resistance, and HOMA-B assesses insulin secretion dysfunction^27^. The HOMA-IR index is the product of basal glucose and insulin levels divided by 22.5; the HOMA-B score is computed as the product of 20 and basal insulin levels divided by the value of basal glucose minus 3.5^39^.

### T2DM novel subgroups

For T2DM patient classification, first, we excluded T2DM patients at baseline who self-reported a T2DM diagnosis. By using BMI, age at diagnosis, HOMA-B, HOMA-IR, and HbA1c levels, which are measured at the time of diagnosis of T2DM, following previous methods^14^, we conducted a data-driven k-means cluster analysis of 1322 T2DM patients. Before clustering, all variables were converted to a mean value of 0 and a standard deviation (SD) of 1. All extreme outliers greater than 5 SDs from the mean were excluded. We used the elbow method for the number of clusters k to capture the point at which the within-cluster sum of squares rapidly decreased. We used the scikit-learn package in Python version 3.9.7 to conduct K-means cluster analysis.

### Construction of the prediction models

We determined the relationship between the PRS and T2DM incidence based on a multivariate Cox regression model incorporating sex and age (model_1_), which can be represented as,

1 $${model}_{1}: T2DM \sim PRS+\boldsymbol{ }sex+age$$

We calculated the time from the baseline age to the diagnosis of incident T2DM (case) or to the last follow-up age for each person without T2DM (censored). To increase the performance accuracy, we also considered traditional risk factors for T2DM, such as family history, physical measurements, and clinical risk factors, which were measured or answered at baseline. The characteristics of the study population at baseline are presented as means $\pm$ SDs or percentages (Table 1 and Supplementary Table S6). The three different prediction models are represented as

2 $${model}_{2}: T2DM\sim PRS+\boldsymbol{ }sex+age+Family \; History$$

3 $${model}_{3}: T2DM \sim PRS+sex+age+Family\, History+ BMI+ SBP+Smoking \; Status$$

4 $${model}_{4}: T2DM \sim PRS+sex+age+Family \; History+BMI+ SBP+Smoking \; Status+HDL+LDL+TG$$

T2DM: Type 2 Diabetes Mellitus; PRS: Polygenic Risk Score; BMI: Body Mass Index; SBP: Systolic Blood Pressure; HDL: High-Density Lipoprotein; LDL: Low-Density Lipoprotein; TG: Triglyceride.

We corrected the LDL level for using lipid-lowering drugs by dividing the LDL concentration by 0.7^40^ and adjusted the SBP for treated individuals using blood pressure-lowering medication by adding 15 mmHg to the measurements^41^. We excluded DBP from the prediction models because of its strong association with SBP to streamline the model and prevent multicollinearity issues. We evaluated the ability of Harrel’s C-index to predict the performance of the model without PRS to determine the contribution of PRS to predicting T2DM. We excluded fasting glucose and HbA1c levels in the prediction model because these levels are used to diagnose T2DM.

### Statistical analysis

We used sex as a predictor for all Cox regression analyses. We classified participants into three groups according to the percentile of PRS: the top (> 90%) and bottom deciles (< 10%) and the middle (10–90%). The largest 10–90% bin was used as the reference. We also evaluated the standardized PRS, which is a continuous-scale PRS with a mean of zero and a variance of one. Age at T2DM diagnosis was defined as the age at which the patient was diagnosed with T2DM earlier in the survey or the age at the first follow-up meeting the diagnostic criteria. The Schoenfeld residual test was used as a statistical test for the proportional hazards assumption.

### T2DM cumulative prevalence

Cox regression was used to model the time to diagnosis of T2DM, where time was defined as the T2DM diagnosis age for T2DM patients (case) or the last follow-up age for each participant without T2DM (censored).

### T2DM risk progression analysis

Cox regression was used to model development from nondiabetes to T2DM, NGT to prediabetes, and prediabetes to T2DM. We calculated the time from the baseline age to the diagnosis age for incident prediabetes/T2DM (case) or to the last follow-up age for each person without incident prediabetes/T2DM (censored).

### T2DM severity progression analysis

We used the same Cox regression model for insulin prescription and T2DM complications. The time was calculated from the age at T2DM diagnosis to the age at which the patient answered incident insulin prescription/T2DM complications (case) or the age at last follow-up (censored). Because none of the individuals in the bottom-decile PRS group were prescribed insulin, we classified participants into two groups: the top (> 90%) and the remaining (90%) PRS groups. We used the remaining PRS group as the reference.

### HOMA analysis

We calculated the median and confidence interval (CI) of the HOMA-H index at each time point for the top-decile PRS group and the remaining group. To evaluate whether the HOMA retrograde trajectories between the top-decile PRS group and the remaining group were significantly different, we first obtained the test statistic as the summation of the median difference between the two groups at each time point. We performed a permutation test to obtain the p value. We randomly sampled the PRS group index 10,000 times and calculated the same test statistic for each permuted sample. Permutation p values were calculated as the proportion of test statistics from the permuted samples that were more extreme than the observed test statistics.

All the above statistical analyses were conducted using R version 4.0.3 software, and a 2-sided p value < 0.05 was considered to indicate statistical significance.

### Ethics declaration

The study was approved by the Institutional Review Board of Seoul National University (approval number: IRB No. E2012/002-001). This research was conducted following the Declaration of Helsinki. Informed consents were obtained from all the study participants.

### Supplementary Information

Supplementary Information.

Supplementary Table S7.

## Data Availability

Data in this study were from the Korean Genome and Epidemiology Study (KoGES; 4851–302), National Research Institute of Health, Centers for Disease Control and Prevention, Ministry for Health and Welfare, Republic of Korea. UK Biobank data were accessed under the accession number UKB: 45227. BBJ summary statistics used in this study were downloaded from https://pheweb.jp/.

## References

[1] Sun H (2022). IDF Diabetes Atlas: Global, regional and country-level diabetes prevalence estimates for 2021 and projections for 2045. Diabetes Res. Clin. Pract..

[2] Bae JH (2022). Diabetes fact sheet in Korea 2021. Diabetes Metab. J..

[3] Hostalek U (2019). Global epidemiology of prediabetes—Present and future perspectives. Clin. Diabetes Endocrinol..

[4] Lall K, Magi R, Morris A, Metspalu A, Fischer K (2017). Personalized risk prediction for type 2 diabetes: The potential of genetic risk scores. Genet. Med..

[5] Xue A (2018). Genome-wide association analyses identify 143 risk variants and putative regulatory mechanisms for type 2 diabetes. Nat. Commun..

[6] Khera AV (2018). Genome-wide polygenic scores for common diseases identify individuals with risk equivalent to monogenic mutations. Nat. Genet..

[7] Liu W, Zhuang Z, Wang W, Huang T, Liu Z (2021). An improved genome-wide polygenic score model for predicting the risk of type 2 diabetes. Front. Genet..

[8] Mars N (2020). Polygenic and clinical risk scores and their impact on age at onset and prediction of cardiometabolic diseases and common cancers. Nat. Med..

[9] Lyssenko V (2008). Clinical risk factors, DNA variants, and the development of type 2 diabetes. N. Engl. J. Med..

[10] Go MJ (2016). Genetic-risk assessment of GWAS-derived susceptibility loci for type 2 diabetes in a 10 year follow-up of a population-based cohort study. J. Hum. Genet..

[11] Mak TSH, Porsch RM, Choi SW, Zhou X, Sham PC (2017). Polygenic scores via penalized regression on summary statistics. Genet. Epidemiol..

[12] Ge T, Chen CY, Ni Y, Feng YA, Smoller JW (2019). Polygenic prediction via Bayesian regression and continuous shrinkage priors. Nat. Commun..

[13] Moon S (2019). The Korea Biobank Array: Design and identification of coding variants associated with blood biochemical traits. Sci. Rep..

[14] Ahlqvist E (2018). Novel subgroups of adult-onset diabetes and their association with outcomes: A data-driven cluster analysis of six variables. Lancet Diabetes Endocrinol..

[15] Mavaddat N (2019). Polygenic risk scores for prediction of breast cancer and breast cancer subtypes. Am. J. Hum. Genet..

[16] Sharp SA (2019). Development and standardization of an improved type 1 diabetes genetic risk score for use in newborn screening and incident diagnosis. Diabetes Care.

[17] Kim MK (2019). 2019 clinical practice guidelines for type 2 diabetes mellitus in Korea. Diabetes Metab. J..

[18] ElSayed NA (2022). 2. Classification and diagnosis of diabetes: Standards of care in diabetes—2023. Diabetes Care.

[19] Tabak AG, Herder C, Rathmann W, Brunner EJ, Kivimaki M (2012). Prediabetes: A high-risk state for diabetes development. Lancet.

[20] Ashenhurst JR (2022). A polygenic score for type 2 diabetes improves risk stratification beyond current clinical screening factors in an ancestrally diverse sample. Front. Genet..

[21] Huang X, Han Y, Jang K, Kim M (2022). Early prediction for prediabetes and type 2 diabetes using the genetic risk score and oxidative stress score. Antioxidants.

[22] Glechner A (2018). Effects of lifestyle changes on adults with prediabetes: A systematic review and meta-analysis. Prim. Care Diabetes.

[23] American Diabetes, A (2021). 9. Pharmacologic approaches to glycemic treatment: Standards of medical care in diabetes-2021. Diabetes Care.

[24] Yun JS (2022). Polygenic risk for type 2 diabetes, lifestyle, metabolic health, and cardiovascular disease: A prospective UK Biobank study. Cardiovasc. Diabetol..

[25] Tremblay J (2021). Polygenic risk scores predict diabetes complications and their response to intensive blood pressure and glucose control. Diabetologia.

[26] Udler MS (2018). Type 2 diabetes genetic loci informed by multi-trait associations point to disease mechanisms and subtypes: A soft clustering analysis. PLoS Med..

[27] Laakso M (2019). Biomarkers for type 2 diabetes. Mol. Metab..

[28] Hur HJ (2022). Association of polygenic variants with type 2 diabetes risk and their interaction with lifestyles in Asians. Nutrients.

[29] Suzuki K (2019). Identification of 28 new susceptibility loci for type 2 diabetes in the Japanese population. Nat. Genet..

[30] Saisho Y (2015). beta-cell dysfunction: Its critical role in prevention and management of type 2 diabetes. World J. Diabetes.

[31] Hahn SJ, Kim S, Choi YS, Lee J, Kang J (2022). Prediction of type 2 diabetes using genome-wide polygenic risk score and metabolic profiles: A machine learning analysis of population-based 10-year prospective cohort study. EBioMedicine.

[32] Wagner R (2021). Pathophysiology-based subphenotyping of individuals at elevated risk for type 2 diabetes. Nat. Med..

[33] Nam K, Kim J, Lee S (2022). Genome-wide study on 72,298 individuals in Korean biobank data for 76 traits. Cell Genomics.

[34] Zhou W (2020). Scalable generalized linear mixed model for region-based association tests in large biobanks and cohorts. Nat. Genet..

[35] Ishigaki K (2020). Large-scale genome-wide association study in a Japanese population identifies novel susceptibility loci across different diseases. Nat. Genet..

[36] Genomes Project, C. (2015). A global reference for human genetic variation. Nature.

[37] Yang SJ, Kwak SY, Jo G, Song TJ, Shin MJ (2018). Serum metabolite profile associated with incident type 2 diabetes in Koreans: Findings from the Korean Genome and Epidemiology Study. Sci. Rep..

[38] Levey AS (2009). A new equation to estimate glomerular filtration rate. Ann. Intern. Med..

[39] Matthews DR (1985). Homeostasis model assessment: insulin resistance and beta-cell function from fasting plasma glucose and insulin concentrations in man. Diabetologia.

[40] Tobin MD, Sheehan NA, Scurrah KJ, Burton PR (2005). Adjusting for treatment effects in studies of quantitative traits: Antihypertensive therapy and systolic blood pressure. Stat. Med..

[41] Noordam R (2019). Multi-ancestry sleep-by-SNP interaction analysis in 126,926 individuals reveals lipid loci stratified by sleep duration. Nat. Commun..

