# Supplementary material for: The clinical relevance of a polygenic risk score for type 2 diabetes mellitus in the Korean population
Source: Sci Rep. 2024 Mar 8;14:5749. doi: 10.1038/s41598-024-55313-0 (PMC10923897; doi:10.1038/s41598-024-55313-0)
Supplement: Supplementary file 1 — Supplementary Information. [file 41598_2024_55313_MOESM1_ESM.pdf]

Supplementary Materials

for

The Clinical relevance of Polygenic Risk Scores to Type 2 Diabetes Mellitus in Korean Population

Na Yeon Kim[1], Haekyung Lee[2], Sehee Kim[3], Ye-Jee Kim[3], Hyunsuk Lee[4,5], Junhyeong Lee[1],

Soo Heon Kwak[4], Seunggeun Lee[1,*]

Corresponding author

Seunggeun Lee

Graduate School of Data Science

Seoul National University, Seoul, South Korea

lee7801@snu.ac.kr

Supplementary Figures

Supplementary Fig. S1 Manhattan plot of meta-analysis of East Asian T2DM

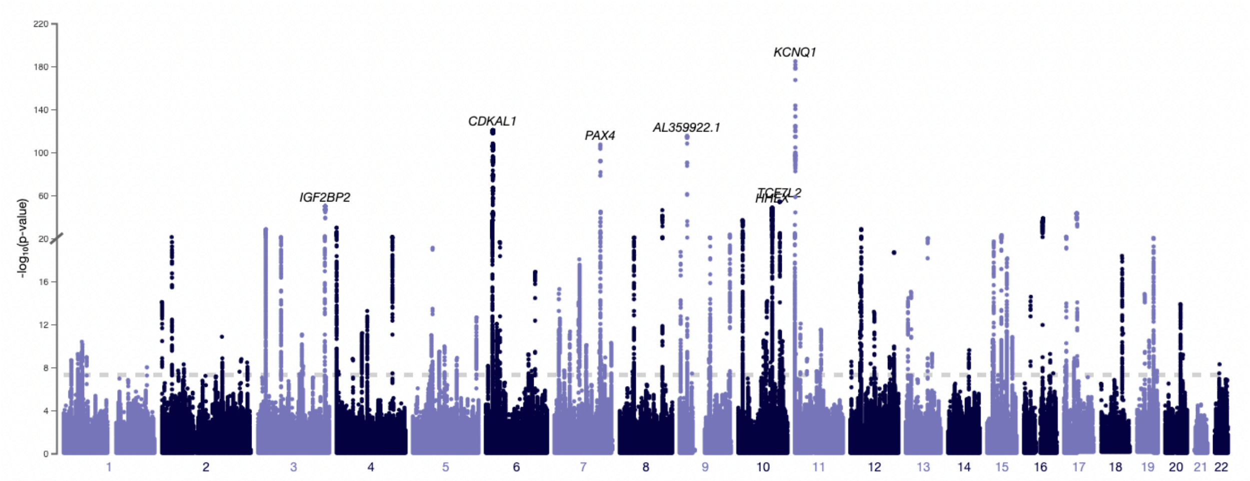

Supplementary Fig. S2 KoGES Ansan and Ansung: Kaplan-Meier curve for cumulative prevalence of T2DM by PRS group

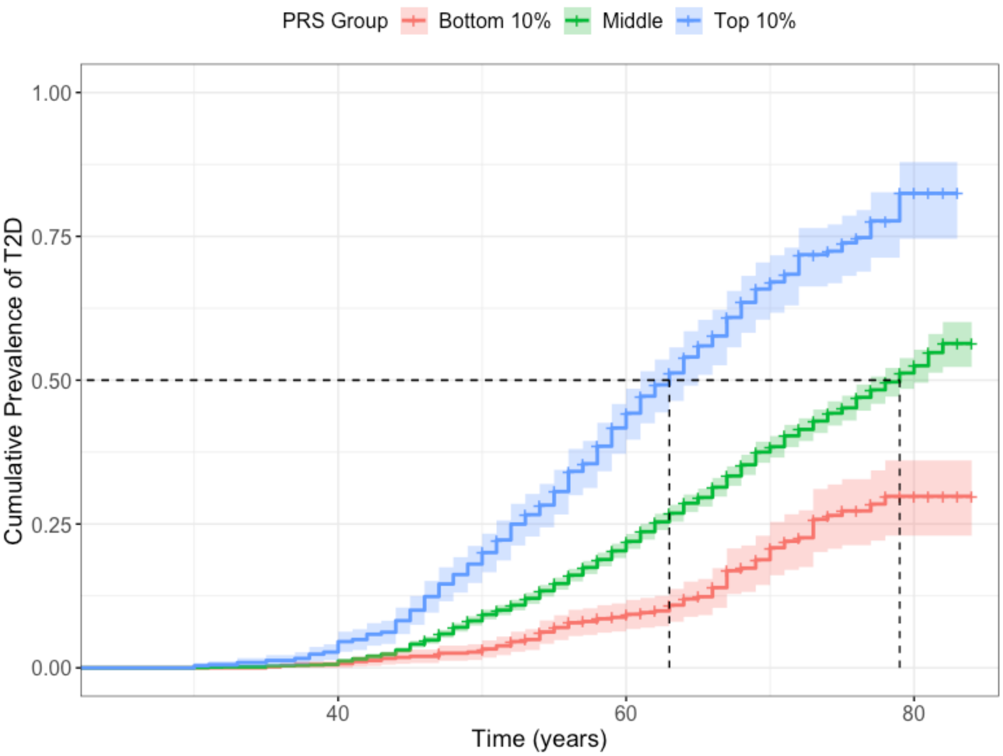

Each shaded area represents 95% confidence band for each curve. Each dash line indicates median age-at-T2DM diagnosis for each PRS group.

Supplementary Fig. S3 UKBB East Asian validation: Kaplan-Meier curve for cumulative prevalence of T2DM by PRS Group

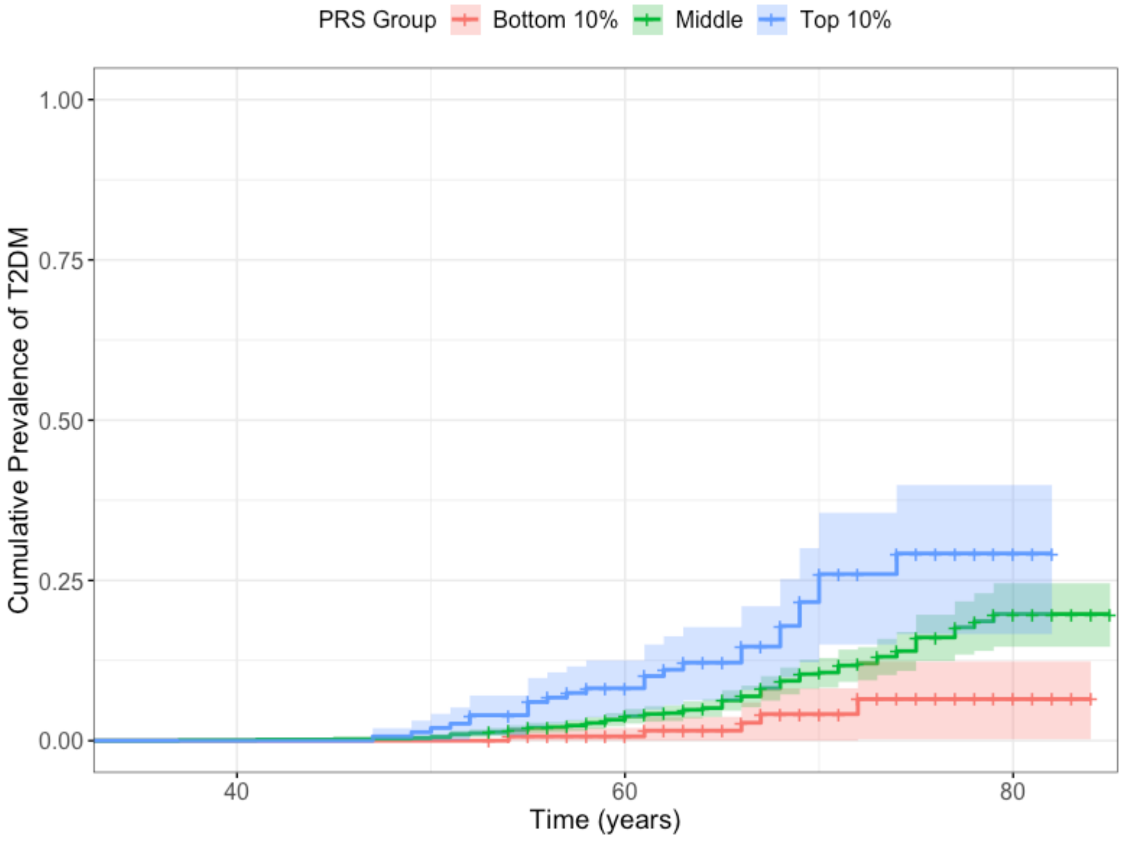

Each shaded area represents 95% confidence band for each curve.

Supplementary Fig. S4 Retrograde trajectory of HOMA-IR and HOMA-B. A. Diabetes (HOMA-IR) B. Non-Diabetes (HOMA-IR) C. Diabetes (HOMA-B) D. Non-Diabetes (HOMA-B)

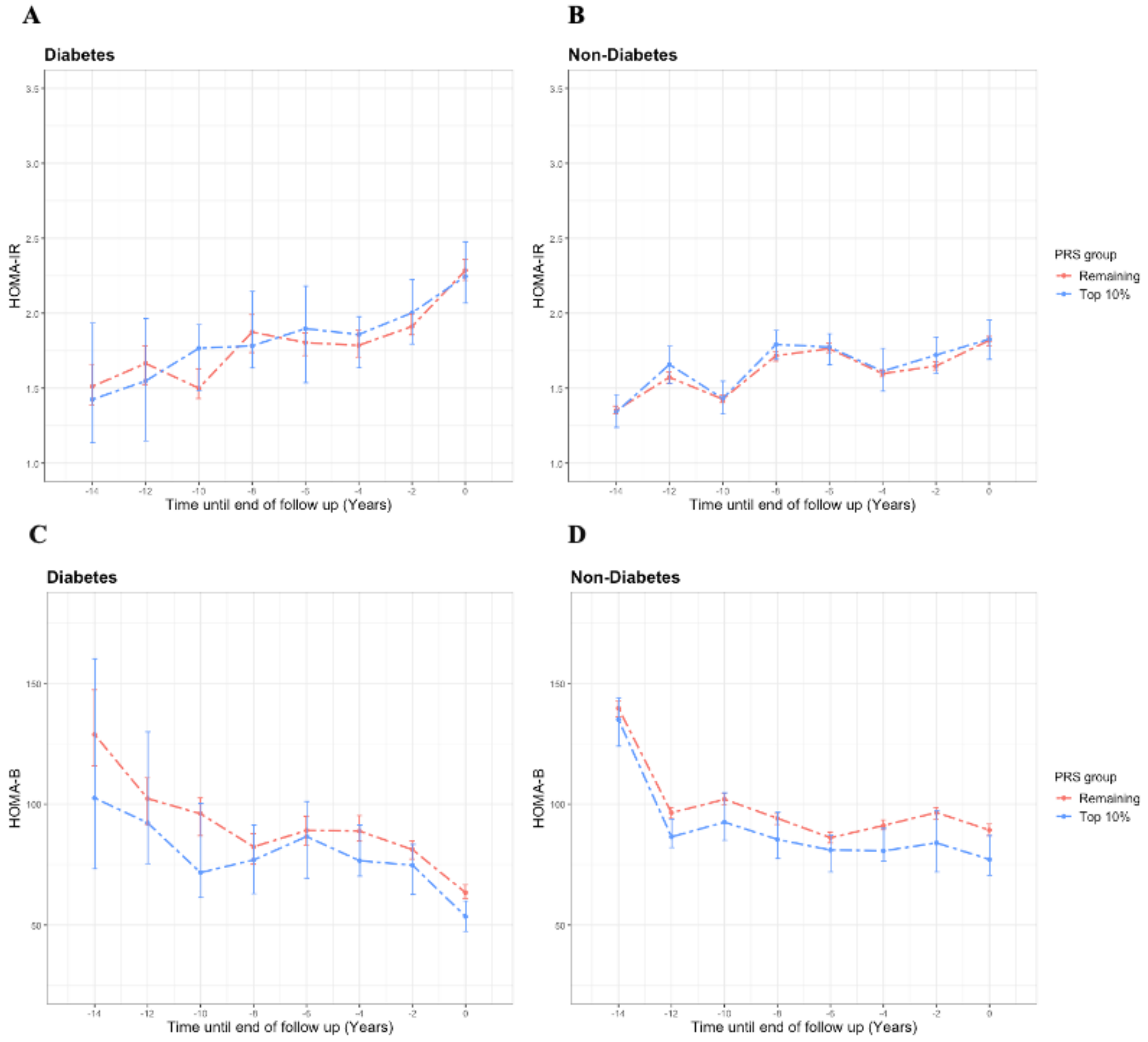

Each bar represents 95% confidence interval at each time point.

Supplementary Fig. S5 Novel diabetic subgroup characteristics

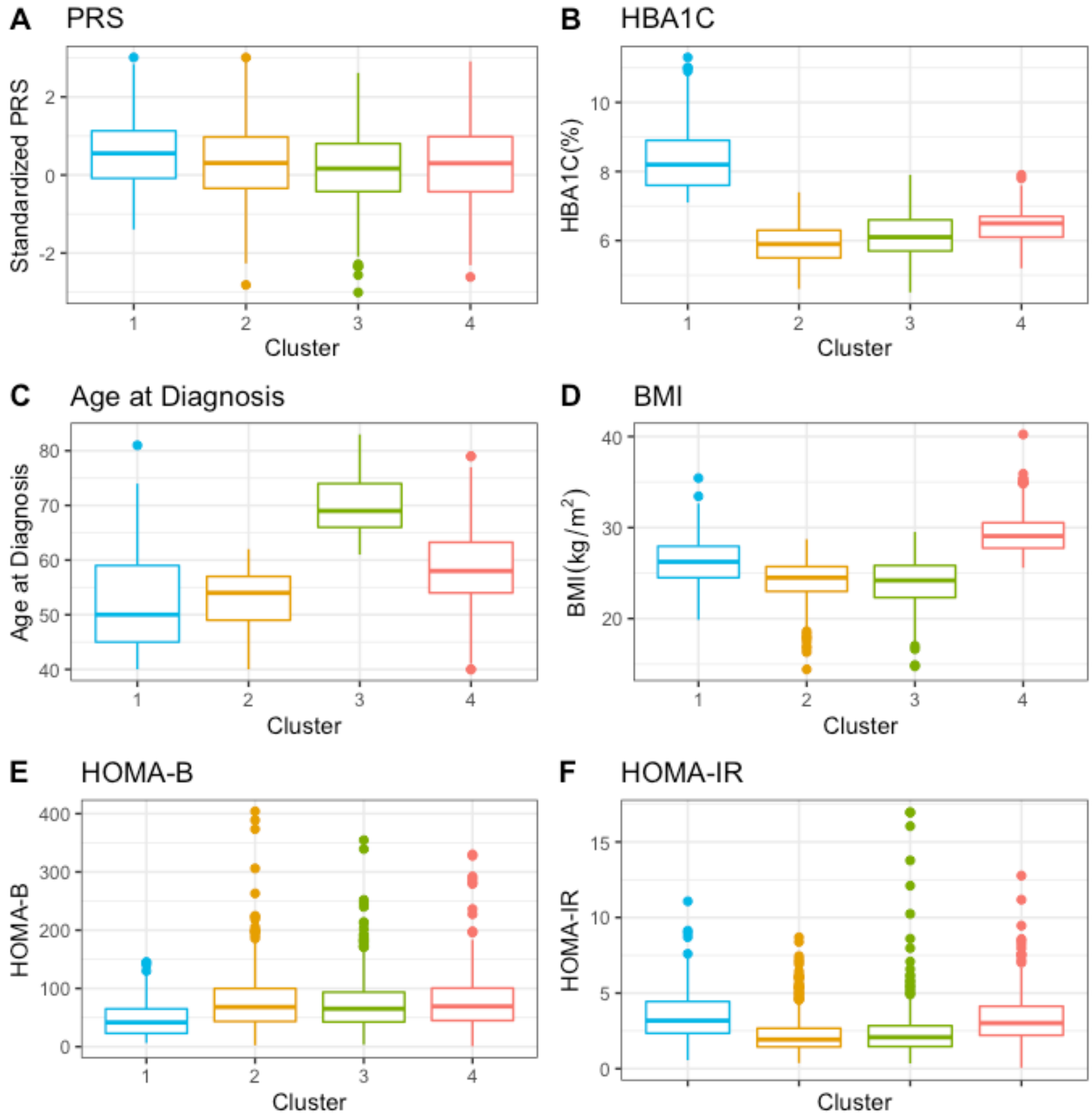

Supplementary Tables

Supplementary Table S1 Area under the receiver operating characteristics curve (AUC) of different methods

	**T2DM ~ PRS**	**T2DM ~ PRS + sex + age**
**Lassosum**	0.64859	0.67599
**PRS-CS**	0.64766	0.67691

Supplementary Table S2 Dropout rate of Participants of Koges_Ansan and Ansung dataset

Follow-up	Baseline	1st	2nd	3rd	4th	5th	6th	7th
**Dropout Rate (%)**	0	5.14	10.66	15.12	14.48	15.45	17.81	11.57

Supplementary Table S3 Cumulative prevalence of T2DM for baseline and each follow-up of Koges_Ansan and Ansung dataset

**Follow-up**	**Baseline**	**1st**	**2nd**	**3rd**	**4th**	**5th**	**6th**	**7th**
**NGT**	2572	2539	1821	1351	975	838	711	600
**Prediabetes**	2172	2015	2544	2832	3043	3039	3048	2995
**Diabetes**	746	936	1125	1307	1472	1613	1731	1895

Supplementary Table S4 Results of Cox regression analysis for cumulative prevalence of T2DM by PRS group

PRS	Hazard Ratio	95% CI	P-value
**Categorized PRS**			
Bottom Decile	0.45	*0.36 – 0.56*	*2.84E-13*
Middle (10-90%)	1.00 (reference)		
Top Decile	2.290	1*2.02 – 2.59*	*2.00E-16*

In the Cox regression model, sex was included as a predictor.

Supplementary Table S5 Results of Cox regression analysis for predicting T2DM complication specified as macrovascular complications and nephropathy

T2DM Complications	PRS	Hazard Ratio	95% CI	P-value
**Macrovascular Complications**	**Categorized PRS**			
	Bottom Decile	0.6047	0.2233 – 1.637	0.322
	Middle (10-90%)	1.00 (reference)		
	Top Decile	0.7980	0.5325 – 1.196	0.275
	**Standardized PRS**	0.9816	0.8415 – 1.145	0.813
**Nephropathy**	**Categorized PRS**			
	Bottom Decile	0.9122	0.4675 – 1.780	0.788
	Middle (10-90%)	1.00 (reference)		
	Top Decile	0.8548	0.6197 – 1.179	0.339
	**Standardized PRS**	0.9177	0.8110 – 1.038	0.173

Macrovascular complications include myocardial infarction, coronary artery disease, congestive heart failure, cerebrovascular disease, peripheral artery disease.

Supplementary Table S6 Characteristics at the baseline of the KoGES_Ansan and Ansung non-diabetic population dataset

	PRS Bottom Decile (n = 348)	Middle (10-90%) (n = 2075)	PRS Top Decile (n = 149)	P-value
**Basic Information**				
Male (%)	173 (49.71)	941 (45.35)	66 (44.30)	0.295
Age (year)	50.73 ± 8.24	49.69 ± 8.46	48.61 ± 7.41	0.014
**Physical Measurements**				
BMI (kg/m^2)	24.08 ± 2.89	24.10 ± 2.87	24.41 ± 2.80	0.424
SBP (mmHg)	117.62 ± 16.03	117.98 ± 17.07	118.74 ± 18.08	0.796
DBP (mmHg)	79.03 ± 10.74	78.85 ± 11.41	79.03 ± 11.85	0.95
**Clinical Risk Factors**				
HDL (mg/dL)	50.07 ± 11.75	50.48 ± 11.69	50.01 ± 11.39	0.764
LDL (mg/dL)	115.64 ± 30.30	115.72 ± 31.63	115.55 ± 29.62	0.997
TG (mg/dL)	133.18 ± 81.57	152.93 ± 111.57	126.77 ± 61.24	0.759
Fasting Glucose (mg/dL)	84.29 ± 11.47	131.97 ± 93.41	87.20 ± 7.00	6.06e-05
Fasting Insulin (μIU/mL)	7.05 ± 3.58	7.29 ± 4.76	7.74 ± 7.61	0.348
HbA1C (%)	5.29 ± 0.32	5.34 ± 0.22	5.34 ± 0.22	0.00719
**Others**				
Current Smoking (%)	89 (25.58)	460 (22.17)	30 (20.13)	0.254
Family History (%)	21 (6.03)	200 (9.64)	15 (10.07)	0.091

BMI: Body Mass Index; SBP: Systolic Blood Pressure; DBP: Diastolic Blood Pressure; HDL: High-Density Lipoprotein; LDL: Low-Density Lipoprotein; TG: Triglyceride. Obtained P-value using one-way ANOVA analysis.
